# A Novel BERT-Based Machine Learning Approach for Enhanced CSF Leak Prediction in Endoscopic Endonasal Skull Base Surgery

**DOI:** 10.1055/a-2719-8970

**Published:** 2025-10-23

**Authors:** Rayan Alfallaj, Yakoub Bazi, Mohamad M. A. Rahhal, Mansour Zuair, Ashwaq Alqurashi, Ahmad Alroqi, Abdulrazag Ajlan, Saad Alsaleh, Abdulaziz S. Alrasheed

**Affiliations:** 1 Department of Otolaryngology - Head and Neck Surgery, College of Medicine, King Saud University, Riyadh, Saudi Arabia; 2 Computer Engineering Department, College of Computer and Information Sciences, King Saud University, Riyadh, Saudi Arabia; 3 Applied Computer Science Department, College of Applied Computer Science, King Saud University, Riyadh, Saudi Arabia; 4 Devision of Neurosurgery, King Saud University, Riyadh, Saudi Arabia

**Keywords:** AI, CSF, machine learning, NLP, skull base, BERT, reconstruction, rhinology

## Abstract

**Objectives:**

To evaluate the performance of the BERT (bidirectional encoder representations from transformers) model in predicting cerebrospinal fluid (CSF) leaks and compare it with traditional logistic regression analysis.

**Methods:**

In this study, we employed a machine learning-based natural language processing (NLP) model, specifically BERT, and compared its performance to conventional statistical logistic regression in predicting CSF leaks. We analyzed all cases of skull base pathologies treated by a multidisciplinary team specializing in rhinology and skull base surgery, and neurosurgery at a single center between March 2015 and July 2020. The dataset included the following factors: (1) demographics, (2) perioperative clinical CSF leak indicators, (3) pathology-related factors, (4) surgical factors, and (5) perioperative CT scan features.

**Results:**

The BERT model outperformed the traditional logistic regression model in predicting CSF leaks, achieving an AUC of 1.0000, sensitivity of 1.0000, specificity of 0.9808, positive predictive value (PPV) of 0.8889, negative predictive value (NPV) of 1.0000, and an F1 score of 0.9657. In contrast, the logistic regression model yielded an AUC of 0.847, with a sensitivity of 0.2143, specificity of 0.9060, PPV of 0.4471, and NPV of 0.7649.

**Conclusion:**

BERT NLP model outperforms traditional logistic regression in predicting cerebrospinal fluid leaks after endoscopic skull base surgery, demonstrating superior accuracy with qualitative clinical data, enhancing risk stratification and decision-making.

## Introduction

The endoscopic endonasal approach has become the dominant and widely accepted method for removing skull base tumors in the sellar and parasellar regions. 1 Skull base reconstruction plays a pivotal role in mitigating the risk of cerebrospinal fluid (CSF) leakage and its associated complications, including the development of meningitis. 2 Achieving successful skull base reconstruction and preventing CSF leaks has significant positive implications for reducing hospitalization duration and alleviating the burden on healthcare costs. 3 CSF leak is a recognized risk during endoscopic sellar surgery, especially when managing large tumors or craniopharyngiomas. Evidence indicates that intraoperative CSF leaks occur in about 30.1% of cases, while postoperative leaks are reported in roughly 3.7 to 9%, 4 5 influenced by various factors such as surgeon experience, tumor characteristics, and individual patient-related variables. The risk of CSF leak following endoscopic skull base surgery is further affected by tumor size and consistency, patient age, BMI, and surgical factors 6 ; prevention depends on appropriate reconstruction techniques, with autologous grafts used for small, low-flow defects and vascularized tissue, particularly the nasoseptal flap, serving as the gold standard for larger or high-flow leaks in anterior skull base reconstruction. 7

Despite established reconstruction techniques and known risk factors, accurately predicting which patients will develop postoperative CSF leaks remains challenging, necessitating more sophisticated predictive methodologies.

The advancement of artificial intelligence in healthcare enables the development of machine learning (ML) models that can predict CSF leak risk factors. 7 AI models can integrate multiple patient, tumor, and surgical variables to predict intraoperative CSF leaks. In a cohort of 238 pituitary adenoma cases, machine-learning methods were tested; random forest performed best, accurately identifying most leaks and outperforming traditional statistics and other ML models. 8

This capability will significantly impact surgical practice by informing pre-, intra-, and postoperative management decisions, including reconstruction method selection. In natural language processing (NLP), BERT has become a benchmark model because it is first exposed to vast amounts of raw, unlabeled text and can process information from both directions across relatively long passages. BERT, short for bidirectional encoder representations from transformers, uses only the encoder part of the transformer architecture to learn word meanings that depend on the words that come before and after them, producing a highly adaptable, context-aware language representation. Although the original BERT was trained on general-purpose English text, researchers have since built tailored versions for specialized domains. For example, BioBERT, BlueBERT, SciBERT, and PubMedBERT have been retrained on biomedical literature so the model recognizes technical vocabulary and clinical phrasing more accurately. These domain-specific variants preserve BERT's key strengths, bidirectional context, and the ability to digest long sequences while improving performance on biomedical text-mining tasks. 9 This is the first study to compare a BERT-based model with conventional statistical techniques for detecting CSF-leak risk factors in endoscopic skull-base surgery, leveraging qualitative (nonnumeric) inputs to simplify the handling of large, variable datasets and ultimately boost predictive accuracy and guide reconstruction decisions more effectively.

## Materials and Methods

**Fig. 1:**
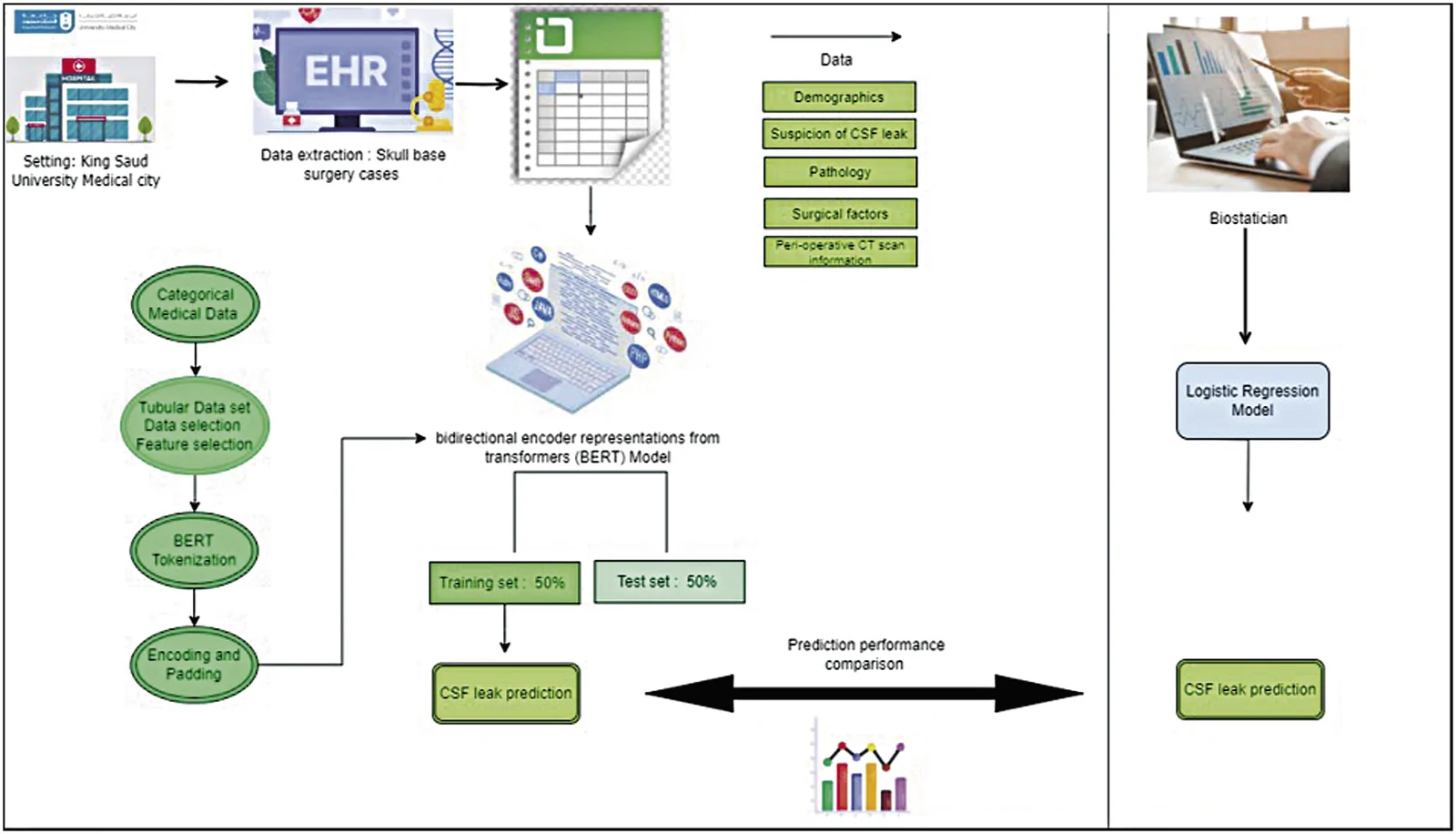
Methodological overview of the study process.

King Saud University Medical City, in collaboration with the College of Computer Science at King Saud University. In this study, we developed a traditional logistic regression model and compared its performance to an NLP model, specifically a BERT model. Ethical approval for the study was obtained from the ethics committee at the College of Medicine at King Saud University (no. 23-8232). Fig. 1 shows the overall methodological process of this project.

### Data Categorization

We extracted information from healthcare records within the study setting, including all cases of skull base pathologies managed by a multidisciplinary team specializing in rhinology, skull base surgery, and neurosurgery at King Saud University Medical City. The available data, which served as input for both the BERT model and the traditional statistical model, were categorized into the following groups: demographics: age, gender, and BMI. Perioperative clinical CSF leak indicators: postoperative clinical symptoms or signs raising suspicion, need for postoperative CT scans and their timing, and length of hospital stay. Pathology-related factors: tumor size, presence of hydrocephalus, tumor location, and histopathology. Surgical factors: type and extent of surgical approach, preoperative CSF diversion, intraoperative CSF leak presence, CSF leak flow rate, nasal flap reconstruction, use of nasal packing, and types of reconstruction materials used. Perioperative CT scan features, including the timing, presence, and location of pneumocephalus; the presence and size of any hematoma; graft characteristics; signs of air communication; and findings from repeat CT scans, were evaluated by a skull-base team comprising a rhinologist, neurosurgeon, and radiologist. The resulting radiological findings data were then entered manually into a text-based datasheet.

### Development of the Traditional Logistic Regression Model

Descriptive statistics were generated using the compareGroups package, with missing data imputed (Hmisc), categorical variables factorized, and class imbalance addressed via upsampling (caret). Continuous data were summarized as mean ± SD or median/IQR, and categorical data as counts and percentages. Univariate analysis used Somers' D and the C statistic (AUC) to assess variable discrimination. Clinically relevant variables with high C statistics were included regardless of significance. Upsampling outperformed SMOTE and ROSE, preserving data structure and improving model performance. A bootstrap approach reduced overfitting, and nonsignificant variables were excluded. The final logistic regression model, validated by ROC and kappa statistics, showed improved predictive accuracy and robustness.

### Development of the BERT Model

Categorical medical data input: previously described were collected as input data, forming the foundation of the predictive model.

### Tabular Dataset

Each patient record is represented as a sequence *x* *=* *[c _1_ ,…,c⅞,y]* , where *c* contains *k* categorical attributes encoded as their text labels (e.g., “male,” “Craniopharyngioma”) and *y∈{0,1}* denotes the presence of a CSF leak. The entire text string is passed to the standard BERT tokenizer (bert-base-uncased), which automatically converts the words into WordPiece tokens, adds the special *[CLS]* and *[SEP]* markers, and pads or truncates the sequence to a fixed length.

**Fig. 2:**
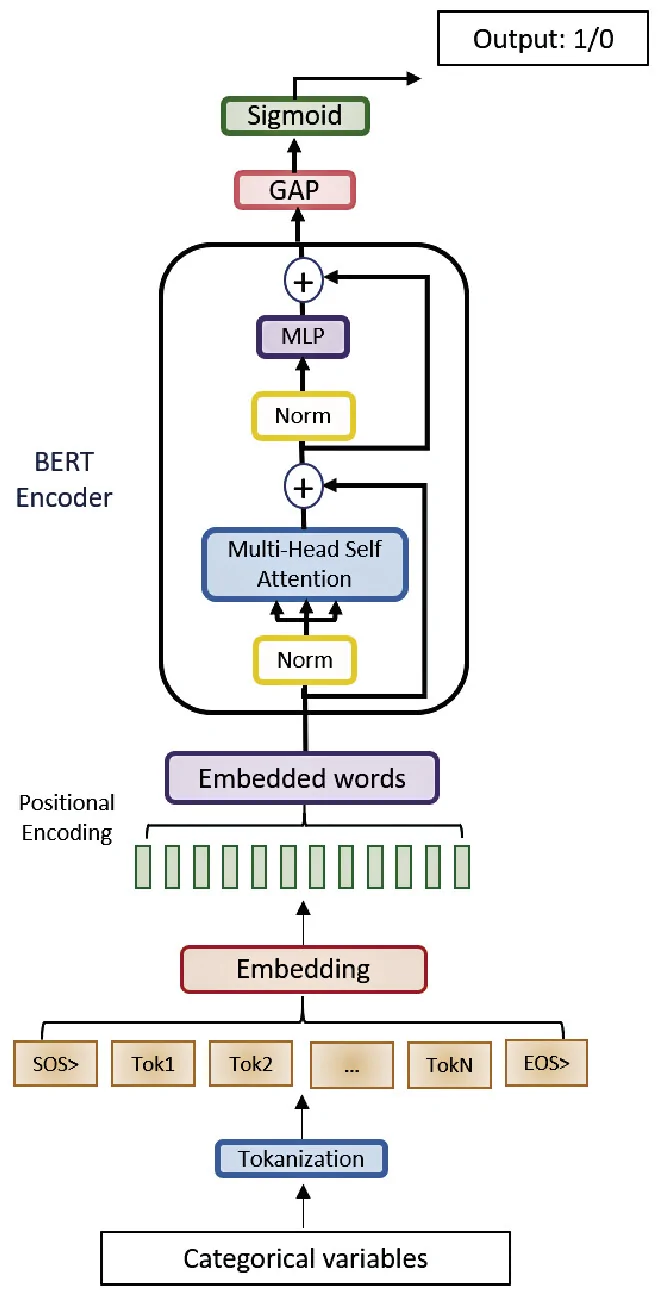
Overview of the classification pipeline based on BERT for CSF leak detection.

The resulting token IDs are fed to the BERT encoder. Within each layer, multi-head self-attention computes contextualized representations using the canonical (see Supplementary Material S1 , available in the online version only, for formulas details), followed by residual connections, layer normalization, and feed-forward sublayers. After the final layer, the [CLS] token is prepended to each sequence, and its final hidden state is extracted as a pooled representation and passed through a linear layer to classify CSF leak versus no leak, as illustrated in Fig. 2 .

All mathematical derivations, hyperparameters, and the training loss are presented in Supplementary Material S1 (available in the online version only).

### BERT Model Training and Optimization

The BERT classifier was fine-tuned on twofold cross-validation (50% training/50% testing). Optimization used AdamW (learning-rate 1 × 10 ^−5^ , weight-decay 1 × 10 ^−2^ ) for 20 epochs and a binary cross-entropy objective; focal weighting was applied to mitigate class imbalance (see Supplementary Material S2 , available in the online version only, for the exact loss formula). Performance was reported as the mean of the two folds using accuracy, precision, recall (sensitivity), positive and negative predictive values, F1 score (the harmonic mean of precision and recall, ranging from 0 to 1), and AUC.

## Results

### Descriptive Statistics for the Study Subjects

A total of 116 skull-base surgery patients were included. No statistically significant differences were found between the CSF-leak and no-leak groups in age (median: 29 years vs. 42 years, *p* = 0.526), gender (60.0% vs. 57.4% female, *p* = 1.000), preoperative CSF diversion (53.3% vs. 50.5%, *p* = 1.000), or hydrocephalus (26.7% vs. 8.9%, *p* = 0.065). Weight, tumor location, pathology size, reconstruction materials (fat, fascia, hard graft, flap, nasal pack, glue, gasket), and surgical approach also showed no significant differences. Moreover, within both the gross-total resection (GTR) and subtotal resection (STR) groups, the proportions of CSF-leak versus no-leak cases were not significantly different (all *p* > 0.05) as detailed in Table 1 .

**Table 1:** Descriptive statistics for the study sample

	No CSF leak	CSF leak	*p* -Value
	*n* = 101	*n* = 15	
Age	42.0 [24.0; 54.0]	29.0 [23.5; 49.0]	0.526
Gender			1.000
Female	58 (57.4%)	9 (60.0%)	
Male	43 (42.6%)	6 (40.0%)	
Preoperative diversion			1.000
No	51 (50.5%)	7 (46.7%)	
Yes	50 (49.5%)	8 (53.3%)	
Preoperative hydrocephalus			0.065
No	92 (91.1%)	11 (73.3%)	
Yes	9 (8.91%)	4 (26.7%)	
Weight			
Underweight	5 (4.95%)	0 (0.00%)	1.000
Normal	31 (30.7%)	5 (33.3%)	1.000
Overweight	21 (20.8%)	6 (40.0%)	0.112
Obese	44 (43.6%)	4 (26.7%)	0.338
Location (anterior skull base)			0.580
No	52 (51.5%)	6 (40.0%)	
Yes	49 (48.5%)	9 (60.0%)	
Location (posterior fossa)			0.633
No	92 (91.1%)	13 (86.7%)	
Yes	9 (8.91%)	2 (13.3%)	
Location (sellar/suprasellar)			0.413
No	59 (58.4%)	11 (73.3%)	
Yes	42 (41.6%)	4 (26.7%)	
Size of the pathology			
(1–2) cm	16 (15.8%)	3 (20.0%)	0.710
(2–3) cm	28 (27.7%)	4 (26.7%)	1.000
>3 cm	44 (43.6%)	8 (53.3%)	0.666
Type of reconstruction material			
Fat	77 (76.2%)	13 (86.7%)	0.515
Fascia	34 (33.7%)	6 (40.0%)	0.849
Hard (bone)	42 (41.6%)	10 (66.7%)	0.122
Naso-septal flap	65 (64.4%)	11 (73.3%)	0.695
nasal pack	74 (73.3%)	9 (60.0%)	0.358
Glue	67 (66.3%)	10 (66.7%)	1.000
Gasket	21 (20.8%)	3 (20.0%)	1.000
Endoscopic approach			0.238
Extended approach	61 (60.4%)	12 (80.0%)	
Standard approach (transsphenoidal transsellar)	40 (39.6%)	3 (20.0%)	
GTR (gross total resection)			0.532
No	53 (52.5%)	6 (40.0%)	
Yes	48 (47.5%)	9 (60.0%)	
STR (subtotal resection)			1.000
No	64 (63.4%)	10 (66.7%)	
Yes	37 (36.6%)	5 (33.3%)	

### Performance and Results of Logistic Regression Model

#### Odds Ratio of Different Variables

Preoperative hydrocephalus (odds ratio [OR] = 5.15, *p* = 0.016), overweight status (OR = 7.15, *p* < 0.001), fat use in reconstruction (OR = 19.65, *p* < 0.001), and gross total resection (OR = 3.83, *p* = 0.001) were all associated with increased odds of CSF leak. In contrast, nasal pack (OR = 0.03, *p* < 0.001), gasket use (OR = 0.20, *p* = 0.003), and sellar/suprasellar tumor location (OR = 0.10, *p* < 0.001) significantly reduced leak risk as shown in Table 2 .

**Table 2:** Logistic regression analysis results

Dependent variable: CSF leak	Odds ratios	Confidence interval	*p* -Value
(Intercept)	0.11	0.03–0.40	0.001
Preoperative hydrocephalus	5.15	1.46–21.61	0.016
Overweight	7.15	2.98–18.60	<0.001
Nasal pack	0.03	0.01–0.10	<0.001
Gasket	0.20	0.07–0.56	0.003
Fat	19.65	5.52–82.96	<0.001
Tumor location (sellar/suprasellar vs. other)	0.10	0.03–0.29	<0.001
Gross total resection (GTR vs. other)	3.83	1.77–8.79	0.001
Observations	202		
*R*^2^ Tjur	0.401		

Intraoperative CSF leaks occurred in 20% of the leak group versus 16.8% of the nonleak group ( *p* = 0.721). Among leak cases, high-flow leaks were more common (13.3% vs. 5.95%), while low-flow leaks were less frequent (6.67% vs. 14.3%) compared to nonleak cases ( *p* = 0.367). Reconstruction methods varied between groups but showed no significant difference ( *p* = 0.167). Postoperative CT revealed significantly more and higher-grade pneumocephalus in the leak group ( *p* = 0.004). Excessive fat was more frequent (58.3% vs. 12.7%, *p* = 0.001), and fat size was more often inadequate ( *p* = 0.048) in the leak group. Hematoma requiring evacuation was slightly more common but not significant ( *p* = 0.492). Postoperative CT revealed factors associated with CSF leaks, including greater extra-cavitary fat graft displacement ( *p* = 0.002) and less favorable solid reconstruction positioning ( *p* = 0.047). Flap adherence was reduced in the leak group ( *p* = 0.060), and air continuity between the nasal and surgical cavities was observed in only one leak case ( *p* = 0.153). Although resection extent varied: standard approach: transsphenoidal transsellar versus Extended approach, the difference was not statistically significant ( *p* = 0.862) as shown in Table 3 .

**Table 3:** Intraoperative and postoperative findings

	No leak	Leak	*p* -Value overall	*n*
	*n* = 101	*n* = 15		
Presence of Intraoperative CSF leak			0.721	116
No	84 (83.2%)	12 (80.0%)		
Yes	17 (16.8%)	3 (20.0%)		
If yes, the CSF flow was			0.367	99
High flow		2 (13.3%)		
Low flow		1 (6.67%)		
None	(100%)	12 (80.0%)		
Type of solid reconstruction			0.167	52
Bone	5 (11.9%)	3 (30.0%)		
Medpore	34 (81.0%)	6 (60.0%)		
Mesh titanium	1 (2.38%)	0 (0.00%)		
Omnipore plate	0 (0.00%)	1 (10.0%)		
Plastic plate	2 (4.76%)	0 (0.00%)		
Presence of pneumocephalus in the postoperative CT scan			0.004	86
Grade 0 (none)	42 (57.5%)	2 (15.4%)		
Grade 1 (dots [<1 mm of air])	3 (4.11%)	0 (0.00%)		
Grade 2 (bubbles [<1 cm of air])	9 (12.3%)	1 (7.69%)		
Grade 3 (1–3 cm air)	4 (5.48%)	4 (30.8%)		
Grade 4 (>3 cm of air)	15 (20.5%)	6 (46.2%)		
Presence of a big hematoma: a need for evacuation?			0.492	85
No	69 (95.8%)	12 (92.3%)		
Yes	3 (4.17%)	1 (7.69%)		
Presence of excessive fat			0.001	83
No	62 (87.3%)	5 (41.7%)		
Yes	9 (12.7%)	7 (58.3%)		
Size Fat-graft?			0.048	82
All fat outside the surgical cavity	0 (0.00%)	1 (7.69%)		
Part of the fat is outside	3 (4.35%)	2 (15.4%)		
Good size	66 (95.7%)	10 (76.9%)		
The location of fat graft?			0.002	77
started to go outside the surgical cavity	9 (13.8%)	4 (33.3%)		
Within the sinus	0 (0.00%)	2 (16.7%)		
Within the surgical cavity	56 (86.2%)	6 (50.0%)		
Sign of air continuity between the surgical cavity and the nasal cavity			0.153	85
No	72 (100%)	12 (92.3%)		
Yes	0 (0.00%)	1 (7.69%)		
Solid reconstruction, is it in a good location?			0.047	64
No	20 (37.7%)	8 (72.7%)		
Yes	33 (62.3%)	3 (27.3%)		
Is the location of the septal flap adherent around all bone defects?			0.060	73
No	14 (22.6%)	6 (54.5%)		
Yes	48 (77.4%)	5 (45.5%)		

Reconstruction materials, including fat, fascia lata, hard grafts, nasoseptal flap, nasal packing, and bone, varied between CSF leak and nonleak groups, but none showed statistically significant differences (all *p* > 0.05), as shown in Table 1 . Histopathological tumor types showed no significant association with CSF leak incidence, with pituitary adenoma being the most common in leak cases (26.7%), followed by meningioma (20.0%), chordoma and craniopharyngioma (13.3% each), and all comparisons yielded nonsignificant *p* -values as shown in Table 4 .

**Table 4:** Histopathology type and CSF leak

Histopathology diagnosis	No leak	Leak	*p* -Value overall
	*n* = 101	*n* = 15	
Atypical cartilaginous neoplasm	1 (0.99%)	0 (0.00%)	1.000
Chordoma	7 (6.93%)	2 (13.3%)	0.328
Craniopharyngioma	6 (5.94%)	2 (13.3%)	0.276
Dermoid cyst	1 (0.99%)	0 (0.00%)	1.000
Epidermoid cyst	0 (0.00%)	1 (6.67%)	0.129
Fibrous dysplasia	2 (1.98%)	0 (0.00%)	1.000
Fungal sinusitis	4 (3.96%)	0 (0.00%)	1.000
Germ cell tumor	1 (0.99%)	0 (0.00%)	1.000
Juvenile nasopharyngeal angiofibroma	3 (2.97%)	0 (0.00%)	1.000
Meningioma	24 (23.8%)	3 (20.0%)	1.000
Meningoencephalocele	3 (2.97%)	1 (6.67%)	0.430
Pilocytic astrocytoma	2 (1.98%)	1 (6.67%)	0.342
Pituitary Adenoma	43 (42.6%)	4 (26.7%)	0.374
Rathke	1 (0.99%)	0 (0.00%)	1.000
Sarcoma	0 (0.00%)	1 (6.67%)	0.129
Spontaneous CSF leak	2 (1.98%)	0 (0.00%)	1.000
Temporal bone fracture	1 (0.99%)	0 (0.00%)	1.000

### Logistic Regression Model Performance

The logistic regression model achieved an AUC of 0.847, with high specificity (90.6%) but low sensitivity (21.4%) and a Kappa of 0.1447, indicating slight agreement. McNemar's test ( *p* < 2e-16) showed significant prediction bias; positive predictive value (PPV) was 44.7%, negative predictive value (NPV) 76.5%, with CSF leak prevalence at 26.2%, detection rate 5.6%, and detection prevalence 12.6%. The balanced accuracy of 56.0% reflects poor performance in detecting true CSF leaks due to class imbalance.

### Performance and Results of the BERT Model

In terms of performance metrics for the BERT model at the confusion matrix (50:2), it was found to have an AUC of 1.0000 and an accuracy of 0.9833, a PPV of 0.8889, an NPV of 1.0000, a recall of 0.9808, and an F1 score of 0.9657

**Fig. 3:**
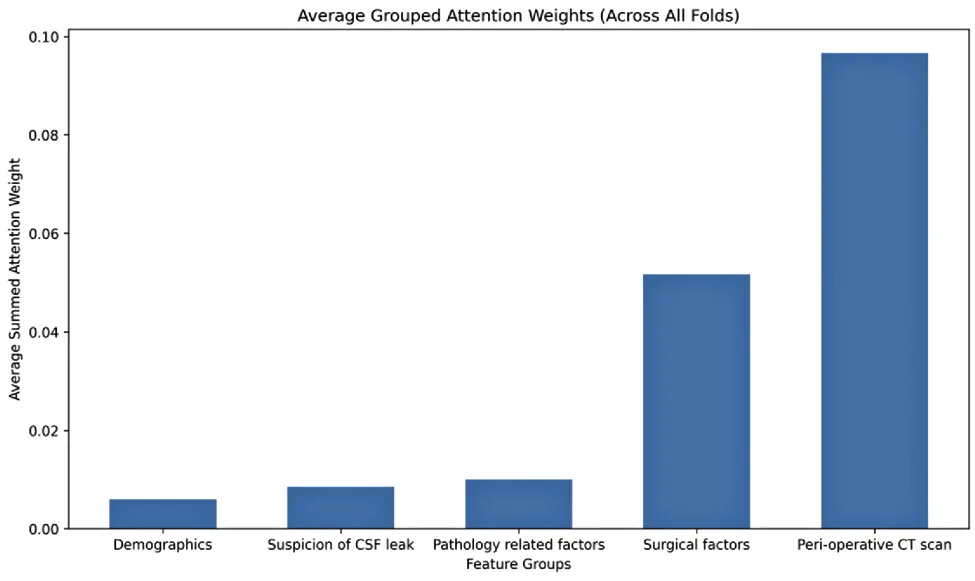
Categorical groups predictors for CSF leak.

The average grouped attention weights across twofold of the proposed model's training revealed the following findings: perioperative CT scans received the highest attention weight by far, followed by surgical factors. The other three groups: demographics, suspicion of CSF leak, and pathology-related factors (including histopathology type) had significantly lower attention weights, as shown in Fig. 3 .

### Comparison of the Logistic Regression Model versus the BERT Model

**Fig. 4:**
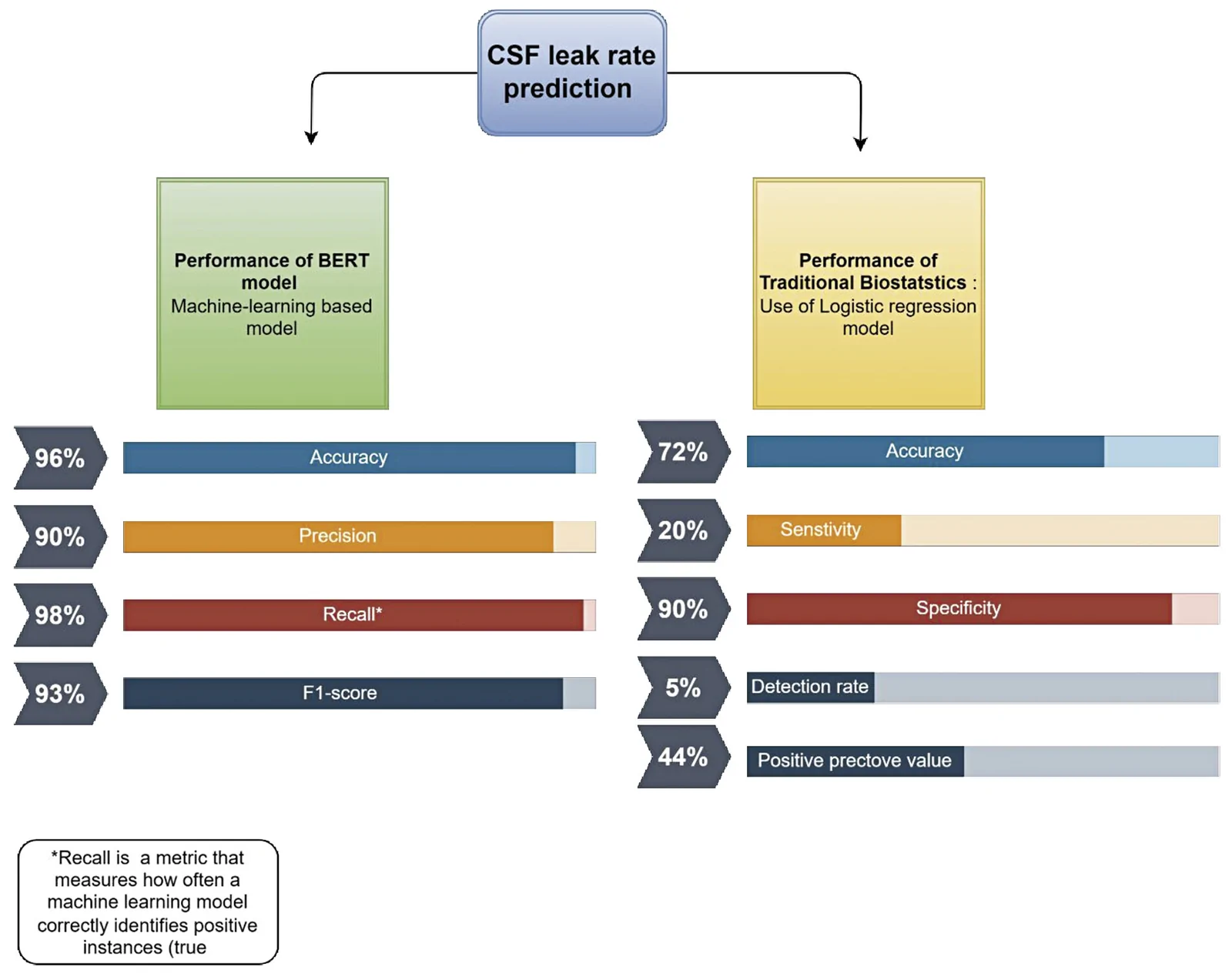
Comparison of performance of the BERT model versus the logistic regression model in the prediction of CSF leak.

The BERT model surpassed the traditional logistic regression model in predicting CSF leaks, achieving an accuracy of 98%, a PPV of 89%, an NPV of 100%, a recall of 98%, and an F1 score of 96%. In contrast, the logistic regression model had an accuracy of 72%, sensitivity of 20%, specificity of 90%, detection rate of 5%, and PPV of 44%, as illustrated in Table 5 and Fig. 4 .

**Table 5:** Comparison of the logistic regression model versus the BERT model

Metric	Logistic regression model	BERT model
AUC	0.847	1.0000
Accuracy	0.725	0.9833
95% CI	(0.7189, 0.731)	
Sensitivity	0.2143	1.0000
Specificity	0.9060	0.9808
Positive predictive value	0.4471	0.8889
Negative predictive value	0.7649	1.0000
F1 score	0.2897	0.9657

## Discussion

In this study, we developed a BERT-based NLP model that transforms qualitative medical data into numerical form for machine learning analysis to predict CSF leaks after skull base surgery. The model significantly outperformed traditional logistic regression, achieving 98% accuracy, 89% precision, 98% recall, and a 96% F1 score, compared to logistic regression's 72% accuracy, 20% sensitivity, 90% specificity, 5% detection rate, and 44% PPV, highlighting the potential of NLP-based models for superior clinical outcome prediction.

Our BERT model analysis revealed that perioperative CT scan features suggestive of CSF leakage carried the highest attention weight for prediction, followed by surgical factors. These findings align with a large multicenter study that evaluated three predictive models (logistic regression, decision tree, and neural network) for CSF leak prediction. Their research identified intraoperative CSF leakage as the most significant risk factor for CSF rhinorrhea, with elevated BMI and revision surgery also contributing significantly in transsphenoidal approaches. 10 The consistency between their conclusions and our results reinforces the superior performance of machine learning methodologies compared to traditional statistical approaches in predicting this critical surgical complication. The BERT model processed the full dataset without manual feature selection, revealing that perioperative CT findings and intraoperative factors were stronger predictors of CSF leak than preoperative variables. Including early postoperative CT data allowed the model to identify key indicators of CSF leak at the time of clinical suspicion, highlighting the value of focusing on perioperative and surgical data for more precise prediction models. A neural network study for predicting intraoperative CSF leaks during pituitary surgery demonstrated 88% classification accuracy (AUC: 0.84), outperforming conventional statistical methods, which identified no significant risk factors. The neural network achieved high sensitivity (83%) and specificity (89%), with high suprasellar Hardy grade, prior surgery, and older age as the primary predictive factors. 11 A systematic review of seven AI studies for CSF leak prediction in pituitary surgery found performance metrics ranging from 0.73 to 0.98 (AUC) and 0.70 to 0.97 (accuracy). Random Forest was the most frequently used algorithm, with k-fold cross-validation as the predominant validation method. Notably, deep learning models demonstrated significantly higher pooled sensitivity than machine learning models (99% vs. 86.2%, *p* < 0.01), while specificity remained comparable between approaches (90.6% vs. 92.1%, *p* = 0.87). 12 Our 12% CSF leak rate aligns with previous studies; elevated BMI was a significant risk factor (OR = 7.15), consistent with Ivan et al, 13 while Fraser et al, 14 specifically identified BMI > 25 kg/m ^2^ and posterior fossa tumors as predictors of higher postoperative leak rates in their 615-patient EEA study. While our data showed no significant correlation between histopathological types or tumor location and CSF leak risk, Zhang et al, 15 found clival tumors associated with higher leak rates in their 100 extra-sellar tumor cases. Our results identified preoperative hydrocephalus as a significant predictor (OR: 5.15, CI: 1.46–21.61, *p* = 0.016), consistent with Patel et al, 16 who found only BMI and hydrocephalus as significant predictors in their 806-case analysis. Our analysis found no statistical significance for various reconstruction techniques, including solid reconstruction. A multicenter analysis of 706 patients reported a 7.8% postoperative CSF leak rate, indicating that rigid reconstruction and older age were protective factors against postoperative sellar leaks, whereas BMI was not linked to increased risk 17 —contrary to our findings. Kuan et al, 18 reported that only intraoperative CSF leak was associated with recurrence in their 300 consecutive repair cases. A large systematic review and meta-analysis of risk factors for postoperative CSF leakage after endonasal endoscopic skull-base surgery reported that overweight and obesity were associated with an OR of 1.88 (95% CI, 1.35 – 2.63; *p* < 0.01), a result that aligns with our findings. Regarding reconstruction, 16 studies (total *n* = 3,579) assessed pedicled vascularized flaps, showing a pooled OR of 0.62 for CSF leakage compared with free grafts. 19 In contrast, our findings demonstrated an unexpectedly high OR of 19.56 for CSF leak when fat grafts were used. This finding diverges from prior literature, in a systematic review of fat graft use in transsphenoidal surgery, postoperative CSF leak requiring intervention was significantly lower in the fat-in-sphenoid-sinus repair group (4.4 %) than in multilayer (20.3 %) or no-repair groups (12.6 %; *p* < 0.01). 20 One plausible explanation for this discrepancy is that fat grafts in our series were preferentially applied to high-flow defects, which inherently carry a greater leak risk.

Our study introduces a novel application of an NLP model for CSF leak prediction in skull base surgery. We believe that the BERT model holds considerable promise for advancing both clinical practice and research. Its implementation in future studies with larger sample sizes may lead to improved clinical decision-making, as the model has demonstrated the ability to identify patterns and associations that traditional statistical methods may overlook. Furthermore, the application of this model in research settings could offer significant advantages in handling large and complex datasets, particularly those involving qualitative variables. Unlike conventional approaches, BERT's key advantage lies in its ability to directly process qualitative data without numerical conversion, preserving contextual information while streamlining analysis. Despite promising results, the exceptionally high-performance metrics suggest potential overfitting or data leakage. As a single-center study, our findings face generalizability limitations. Future validation should include a critical review of CT features, rigorous verification of train/test methodology, validation on independent datasets, and optimization of model complexity. We recommend multicenter implementation of BERT for CSF leak analysis to increase sample size and potentially identify risk factors undetectable through conventional statistical approaches.

## Conclusion

BERT NLP model outperforms traditional logistic regression in predicting CSF leaks after endoscopic skull base surgery, demonstrating superior accuracy with qualitative clinical data, enhancing risk stratification and decision-making.
