# Supplementary material for: A Novel BERT-Based Machine Learning Approach for Enhanced CSF Leak Prediction in Endoscopic Endonasal Skull Base Surgery
Source: J Neurol Surg B Skull Base. 2025 Oct 23;87(5):502–10. doi: 10.1055/a-2719-8970 (PMC13493156; doi:10.1055/a-2719-8970)
Supplement: Supplementary file 1 [file 10-1055-a-2719-8970_29408879.pdf]

Supplementary Material

the dataset be denoted by:

1. $D = \{x_i, x_i\}_{i=1}^{N}$

where x_i represents a vector of categorical features and $y_{i,} \in \{0,1\}$ (with $y_{i,} = 1$ indicating a CSF leak). Each categorical feature vector—representing patient data and CSF leak status—is tokenized using BERT (e.g., *bert-base-uncased*), with special tokens and padding applied to standardize input length, enabling the transformer-based model to generate contextualized embeddings through stacked encoder layers with multi-head self-attention and feed-forward networks. The multi-head self-attention mechanism computes the influence of each token on every other token in the sequence as follows:

$$\text{V2. Attention }(\mathrm{Q}, \mathrm{K}, \mathrm{V}) = \text{softmax }\left(\frac{\mathrm{QK}^{\mathrm{T}}}{\sqrt{d_k}}\right)$$

Here $(\mathrm{Q}, \mathrm{K}, \text{and } \mathrm{V})$ are learned linear transformations of the input embeddings, and d_k is the key dimension. Learned linear transformations are applied to input embeddings, with residual connections and layer normalization ensuring stable training.

BERT model Training and Optimization:

We train our model using the following procedure:

1. Data Splitting: We use 2 folds (50% training and 50% test), then present the Average results**.**

2. Loss Function: We use Binary Cross-Entropy loss, potentially with focal loss to address class imbalance.

3. Optimization: We employ the Adam optimizer with a learning rate scheduler. Given the logits o_i produced by the model for sample i and the corresponding true label y_i the cross-entropy loss is defined as:

$$3. L_{CE} = -\sum_{c=1}^{2} \mathrm{I}(y_i = c) \log \left(\frac{\exp (o_{i,c})}{\sum_{c'} \exp (o_{i,c})} \right)$$

where $\mathrm{I}(.)$ is the indicator function. For a batch of NNN samples, the loss is average:

$$4. L_{CE} = -\sum_{i=1}^{N} L_{CE}(o_i , y_i)$$

4. Training Strategy:

The model is optimized using the AdamW optimizer with the following hyperparameters:

Learning Rate: 1×10−5, **Weight Decay:** 1×10−2 Training is performed for a total of 20. epochs.

5. Evaluation: We assess model performance using metrics such as accuracy, Positive Predictive Value, Negative Predictive Value (NPV), Area Under the Curve (AUC), precision, Sensitivity (Recall), and F1-score.
